# Occupational Health of Frontline Healthcare Workers in the United Arab Emirates during the COVID-19 Pandemic: A Snapshot of Summer 2020

**DOI:** 10.3390/ijerph182111410

**Published:** 2021-10-29

**Authors:** Suad Ajab, Balázs Ádam, Muna Al Hammadi, Najwa Al Bastaki, Mohamed Al Junaibi, Abdulmajeed Al Zubaidi, Mona Hegazi, Michal Grivna, Suhail Kady, Erik Koornneef, Raquel Neves, António Sousa Uva, Mohamud Sheek-Hussein, Tom Loney, Florentino Serranheira, Marília Silva Paulo

**Affiliations:** 1 Institute of Public Health, College of Medicine and Health Sciences, United Arab Emirates University, Al Ain P.O. Box 17666, United Arab Emirates; 200631649@uaeu.ac.ae (S.A.); badam@uaeu.ac.ae (B.Á.); m.grivna@uaeu.ac.ae (M.G.); ekoornneef@gmail.com (E.K.); msheekhussein@uaeu.ac.ae (M.S.-H.); 2 Tawam Hospital, Al Ain P.O. Box 17666, United Arab Emirates; munhammadi@seha.ae; 3 Department of Medical Education and Research, Dubai Health Authority, Dubai P.O. Box 4545, United Arab Emirates; naalbastaki@dha.gov.ae; 4 Ministry of Presidential Affairs, Abu Dhabi P.O. Box 280, United Arab Emirates; aljunaibi.mohamed@gmail.com (M.A.J.); abalzubaidi@mopa.ae (A.A.Z.); sakady@mopa.ae (S.K.); 5 Department of Family Medicine, Mediclinic City Hospital, Dubai P.O. Box 505004, United Arab Emirates; mona.hegazi@mediclinic.ae; 6 Department of Public Health and Preventive Medicine, Second Faculty of Medicine, Charles University, 150 06 Prague, Czech Republic; 7 Health Science Faculty, Higher College of Technology Abu Dhabi, Abu Dhabi P.O. Box 25026, United Arab Emirates; raqui_neves@hotmail.com; 8 CHRC, Comprehensive Health Research Center, Escola Nacional de Saúde Pública, Universidade NOVA de Lisboa, 1600-560 Lisbon, Portugal; asuva@ensp.unl.pt (A.S.U.); serranheira@ensp.unl.pt (F.S.); 9 College of Medicine, Mohammed Bin Rashid University of Medicine and Health Sciences, Dubai P.O. Box 505055, United Arab Emirates; tom.loney@mbru.ac.ae; 10 CHRC, Comprehensive Health Research Center, Nova Medical School, Universidade NOVA de Lisboa, 1169-056 Lisbon, Portugal

**Keywords:** anxiety, burnout, COVID-19, healthcare workers, occupational health, personal protective equipment

## Abstract

The study aim was to understand the availability of personal protective equipment (PPE) and the levels of anxiety, depression, and burnout of healthcare workers (HCWs) in the United Arab Emirates (UAE). This study was an online-based, cross-sectional survey during July and August 2020. Participants were eligible from the entire country, and 1290 agreed to participate. The majority of HCWs were females aged 30–39 years old, working as nurses, and 80% considered PPE to be available. Twelve percent of respondents tested positive for SARS-CoV-2. Half of HCWs considered themselves physically tired (52.2%), reported musculoskeletal pain or discomfort (54.2%), and perceived moderate-to-high levels of burnout on at least one of three burnout domains (52.8%). A quarter of HCWs reported anxiety (26.3%) or depression (28.1%). HCWs reporting not having musculoskeletal pain, having performed physical activity, and higher scores of available PPE reported lower scores of anxiety, depression, and burnout. UAE HCWs experienced more access to PPE and less anxiety, depression, and burnout compared with HCWs in other countries. Study findings can be used by healthcare organizations and policymakers to ensure adequate measures are implemented to maximize the health and wellbeing of HCWs during the current COVID-19 and future pandemics.

## 1. Introduction

Coronavirus disease 2019 (COVID-19), caused by SARS-COV-2, was first detected at the end of 2019 in Wuhan, Hubei province, China [1]. The outbreak was declared an international public health emergency on 30 January 2020 by the World Health Organization (WHO) [2]. In the first two months of 2020, the virus spread to over 76 countries, with 93,091 cases reported by the WHO on the 4th of March [3]. Less than 10 days later, on the 13th of March, the virus had already spread to over 139 countries with more than 145,000 cases and 5400 deaths [4].

The United Arab Emirates (UAE) was the first from the Gulf Cooperation Council countries to report a COVID-19 case on the 29 January 2020 [5,6,7]. Earlier in January, the emergency response system was activated in the UAE, and health authorities started to work on determining gaps and prioritizing workforce, facilities, and availability of both medical devices and personal protective equipment (PPE) [8]. The federal UAE Ministry of Health and Prevention and the National Crisis and Emergency Management Authority, together with the emirate-level health authorities (Department of Health Abu Dhabi, Abu Dhabi Public Health Center, and Dubai Health Authority), worked to amend its licensing processes and procedures for healthcare settings, frontline workers, medical equipment, and technology under a crisis [8].

The COVID-19 pandemic has placed enormous strains on health systems worldwide and healthcare workers (HCWs). In addition to the risk factors related to the COVID-19 pandemic itself, many other aspects of HCW’s health must be considered, such as the impact of psychosocial hazards [9,10]. HCWs, especially physicians and nurses, are exposed to various hazards/risk factors, such as infectious diseases and long working hours, that can lead to work-related mental health issues, including anxiety and burnout [11,12]. Specifically, during a pandemic, they are at greater risk of stress, burnout, and post-traumatic stress disorders [13], potentially leading to a negative impact on the health system due to a decrease in the quality of services provided, a shortage of staff, and increased costs [11,14,15]. Contributory factors of burnout include underlying organic illness, fear of infection and transmitting to a family member, lack of PPE, lack of effective treatment, and the diversity and quantity of information (infodemic) [16,17,18]. Several studies have recently been published on the prevalence of stress, anxiety, depression, and burnout in HCWs during COVID-19 in different hospital settings and countries [19,20,21]. A systematic review and meta-analysis of 12 cross-sectional studies from China and Singapore reported a pooled prevalence of 23.2% and 22.8% for anxiety and depression in HCWs, respectively [22]. 

As far as we know, this study is among the pioneers to address the HCW’s occupational health and wellbeing during the first wave of the pandemic in the UAE. This research may contribute to better planning of the health systems organizations to deal with the allocation of their human resources and medical supplies. It will also contribute to a better understanding of the mental health and fatigue challenges affecting HCWs to help them cope with these unexpected health crises. The study aim was to understand the availability of personal protective equipment (PPE) and the levels of anxiety, depression, and burnout of HCWs in the United Arab Emirates (UAE).

## 2. Materials and Methods

### 2.1. Study Design

This was a cross-sectional study with data collected between July and August 2020. This study is reported according to the Strengthening the Reporting of Observational Studies in Epidemiology (STROBE Statement) [23].

### 2.2. Participants and Setting

All HCWs involved in the care of COVID-19 patients were eligible to participate in this study. The source population was all HCWs in the seven emirates of the UAE: Abu Dhabi, Dubai, Sharjah, Umm Al Quwain, Ajman, Ras Al-Khaimah, and Fujairah. The online survey link was distributed by the administration of the involved healthcare facilities to reach all HCWs within their organization. This strategy aimed to maximize the representativeness of the population sample in the study. The main six healthcare providers involved in treating COVID-19 were contacted to participate in the present study, and four agreed to distribute the survey to their healthcare personnel. Participants needed to give their consent to participate in the study before accessing the study questions.

### 2.3. Data Sources/Measurements (Data Collection Tools)

The data collection tool was an adaptation of the developed Occupational Health Services Survey from Barómetro COVID-19, designed by investigators of the Portuguese National School of Public Health, NOVA University of Lisbon [24]. The survey was translated from Portuguese to English and adapted for the UAE health systems organization. The survey was not validated, but it was piloted in five HCWs before its dissemination, and the results of the pilot are not part of the reported results in our manuscript (Cronbach’s alpha values are reported in the next section).

The data collection tool was organized into three sections:

- In the first section, we asked questions to allow us to characterize the sample by socio-demographic and health and lifestyle (sex, age, specific emirate, sleeping pattern, level of tiredness, and physical activity) and to assess their job context and demands (occupation, healthcare sector, facility type, occupational health and safety, and infection control-related questions, monitoring of SARS-CoV-2 infection, availability and adequacy of PPE);
- In the second section, the Hospital Anxiety and Depression Scale (HADS) [25] was used to understand the perceptions of the HCWs about their anxiety and depression thoughts. The HADS has 14 items: seven related to anxiety and the other seven to depression.
- In the third section, we used the Maslach Burnout Inventory (MBI) [15] questionnaire with its seven-point Likert scale to assess burnout. This questionnaire has 22 items to assess three components of burnout: emotional exhaustion (nine items), depersonalization (five items), and reductions in personal accomplishment (eight items). All the items are scored using a seven-point Likert scale (from 0 (never) to 6 (always)).

### 2.4. Data Analysis

We used descriptive statistical techniques to describe the variables that were considered crucial for the characterization of the occupational health situation of the HCWs in the frontline of COVID-19. Similar to the original questionnaire, occupational health and safety and infection control-related questions, monitoring COVID-19, availability, and adequacy of PPE were asked using six-point Likert scales.

For the HADS analysis, we calculated the sum of each of the 7 questions to anxiety and depression and applied the cutoff of >8 to consider the participant as reporting anxiety or depression, respectively. The internal validity of the anxiety scale (Cronbach’s alpha) was 0.826 and 0.779 for depression.

For each MBI component, we calculated a sum of the score and applied standardized cut-offs for three levels: emotional exhaustion (low: 0–18; moderate: 19–37; high: 38–54), depersonalization (low:0–10; moderate: 11–20; high: 21–30), and personal achievement (low: 34–48; moderate: 17–33; high: 0–16) [26]. For emotional exhaustion and depersonalization, higher scores correspond to higher degrees of experienced burnout, while for personal achievements, lower scores correspond to higher levels of burnout. The Cronbach’s alpha for emotional exhaustion was 0.942, 0.976 for depersonalization, and 0.916 for personal achievement.

The Kolmogorov–Smirnov test was used to assess the normality of the data. Chi-square test and analysis of variance were used to assess the difference in the means between anxiety, depression, and burnout scores and the most relevant socio-demographic characteristics of the HCWs surveyed. Binary and logistic regression analysis was performed to determine potential risk and protective factors for anxiety, depression, and burnout. Crude odds ratio (OR) and adjusted odds ratios (aOR) with 95% confidence intervals (CI) were reported. Statistical significance was defined by a *p*-value ≤ 0.05 and 95% confidence intervals. SPSS 26.0 (SPSS Inc, Chicago, IL, USA) was used to conduct the analysis. The study sample was analyzed as one group due to the study aim of understanding the availability of PPE and the levels of anxiety, depression, and burnout of HCWs in the UAE. This practice is used in similar studies of the same design, although we acknowledge that this is a non-homogeneous group in terms of sex and occupation.

### 2.5. Ethical Considerations

Ethical approval was granted from the Emirates Institutional Review Board for COVID-19 Research (DOH/CVDC/2020/1257), Dubai Scientific Research Committee (DSREC-07/2020_08), the Social Sciences Ethical Committee of the United Arab Emirates University (ERS_2020_6138), and the Institutional Board Research of the involved organizations. 

## 3. Results

### 3.1. Participants

From the 1290 HCWs that agreed to participate in this study, the majority of the study sample were females (78%) and aged 30 to 39 years old (51%) (Table 1). Regarding the number of weekly hours in night shifts, 38.5% had worked less than 9 h, and the same proportion had worked between 9 to 18 h, while 22.9% had worked more than 18 h during the night over the past week. Half of the workers (53.8%) reported having an average sleep of 6–8 h per day, and only 20.2% of them were physically active outside of work (physical activity almost every day or every other day). Half of the HCWs (52.2%) considered themselves physically tired that day (rated 4 or above on a scale from 0 to 6), and 43.8% were more physically tired than last week (rated 4 or above on a scale from 0 to 6).

### 3.2. Job Context and Work Demands

Nurses constituted three-quarters of our sample, and 88% of the HCWs reported working at a publicly funded hospital. General hospitals were the most represented type of facility in our survey, and the vast majority were from the Emirate of Dubai (69%). More than half of participating HCWs (61.4%) were working with COVID-19 patients; out of those, 34.7% were dealing with potential COVID-19 cases in a medical ward, 15.7% in an intensive care unit (ICU), 14.4% in the emergency room, and 9.9% in the COVID-19 screening centers. 

The vast majority of participants (89.6%) were screening themselves daily for COVID-19 symptoms, half of the participants (49.7%) had been suspected to have had contact with a SARS-CoV-2-positive case, and 78.8% of the sample had been tested (by rt-PCR) for SARS-CoV-2 between 1 (32.9%) to 5 times (8.8%). From the total number of participants, half were tested after 72 h of contact with the positive case, but this varied between the first 24 h (17.1%), 24 to 48 h (21.9%), and 48 to 72 h (9.7%). Twelve percent of HCWs tested positive for SARS-CoV-2, but we were not able to determine whether this was community-based transmission or a nosocomial infection.

Occupational health services or infections and prevention control teams managed the risk of infection of HCWs in 95.7% of the healthcare settings, and 69.0% of HCWs had a risk assessment performed. 

Eighty percent of the study population rated the availability of PPEs to be 4 or above (on a scale from 0 to 6). Eighty-two percent considered that the availability of PPE over the weeks of the pandemic improved (rated 4 or above on a scale from 0 to 6), and 82.6% considered PPE to be adequate for their level of exposure (rated 4 or above on a scale from 0 to 6).

### 3.3. Health Effects

#### 3.3.1. Musculoskeletal Symptoms

Half of HCWs (54.2%) reported having more than the normal musculoskeletal pain or discomfort in the past week, and from those, 59.3% identified back pain, 26.1% in their lower body, and 14.5% in their upper limbs. When asked to rate the level of pain from 0 (no pain) to 10 (extreme pain), only a minority of participants reported having average pain (4–6 scores) in the cervical spine (28.7%), low back (34.2%), shoulders (25.9%), and hands, wrists, and arms (21.1%).

#### 3.3.2. Prevalence of Anxiety and Depression

Around a quarter of HCWs scored >8 for anxiety and depression, indicating a prevalence of 26.3% for anxiety and 28.1% for depression.

Anxiety scores showed a non-significant difference by sex, occupation, and healthcare sector (*p* > 0.05). However, differences in anxiety scores were statistically significant by the numbers of hours per night shift (*p* < 0.001), the number of hours of sleep (*p* < 0.001), physical fatigue (*p* < 0.001), physical activity in the past week (*p* < 0.001), musculoskeletal pain (*p* < 0.001), and perceptions on the adequate level of PPE (*p* < 0.001) (Table 2). HCWs reporting no musculoskeletal symptoms, having slept more than 8 h, without intense fatigue, having performed physical activity, and higher scores of available PPE (scored 4 and above) reported lower scores of anxiety.

Depression was not statistically significantly different between males and females nor between private and publicly funded healthcare. Depression varied in terms of occupation (*p* = 0.005), with higher levels of depression reported amongst physicians, HCWs working a greater number of night shift hours (*p* = 0.005), less sleep (*p* < 0.001), more physical fatigue (*p* < 0.001), less physical activity in the past week (*p* < 0.001), more musculoskeletal pain (*p* < 0.001), and a perception of less adequate PPE (*p* < 0.001) (Table 2). HCWs reporting not having musculoskeletal symptoms, having slept more than 8 h, without intense fatigue, having performed physical activity, and higher scores of available PPE (scored 4 and above) reported lower scores of depression.

In the binary logistic regression model (Table 3), factors associated with anxiety and depression are presented. Physicians were more likely to develop anxiety and depression. Higher levels of physical fatigue, musculoskeletal pain, and a lack of physical activity were associated with a greater likelihood of developing anxiety and depression (anxiety: aOR = 12.187, 95% CI: 5.854–25.369; aOR = 1.424, 95% CI: 1.068–1.898; aOR 1.719, 95% CI: 1.111–2.659; depression: aOR = 9.289, 95% CI: 4.236–20.371; aOR = 1.713, 95% CI: 1.276–2.3; aOR: 2.375, 95% CI: 1.499–3.763). HCWs with more than 8 h of sleep in the last week were less likely to suffer from anxiety or depression (anxiety: aOR = 0.404, 95% CI: 0.237–0.689; depression: aOR = 0.45, 95% CI: 0.258–0.785).

#### 3.3.3. Prevalence of Burnout

Half of the HCWs reported moderate-to-high levels of burnout in at least one of the three subcomponents, 52.8% of HCWs reported moderate-to-high levels of emotional exhaustion, 26.6% reported depersonalization, and 30.8% low-to-moderate levels of personal achievement.

There were no statistically significant differences for emotional exhaustion, depersonalization, or personal achievement by sex or healthcare sector (*p* > 0.005). Occupation differed significantly for personal achievement (*p* = 0.004) but not emotional exhaustion or depersonalization. Similar to the factors influencing anxiety and depression, the three domains of burnout differed according to the number of hours per night shift (*p* < 0.005), the number of hours of sleep (*p* < 0.001), physical fatigue (*p* < 0.001), physical activity in the past week (*p* < 0.001), musculoskeletal pain (*p* < 0.001), and perceptions on the adequate level of PPE (*p* < 0.001) (Table 4). 

High levels of emotional exhaustion and depersonalization were reported by HCW working more night shifts, having not performed physical activity, perceiving low PPE availability, and having musculoskeletal pain. Low levels of personal achievement were reported by HCWs working less than 9 h in night shifts, not physically active, perceiving lower availability of PPE, and reporting musculoskeletal pain. 

The regression model adjusted for the adequate level of PPE at the workplace showed that physical activity, sleeping hours, and adequate PPE in the workplace were significantly associated with burnout in HCWS (Table 5). 

## 4. Discussion

Understanding the workload and challenges of frontline HCWs during the COVID-19 pandemic helps implement better occupational health services, infection and prevention control practices, and strategies to help them cope with these maximized work-stress conditions.

### 4.1. Key Findings

Three-quarters of HCWs considered PPE to be readily available and that that availability improved over time. One-quarter of HCWs reported anxiety and depression, and half of them reported high scores in at least one of the domains of burnout (emotional exhaustion, depersonalization, and personal achievement). HCWs reporting not having musculoskeletal pain, having performed physical activity, and higher scores of available PPE reported lower scores of anxiety, depression, and burnout.

### 4.2. Comparison of Findings with Previous Work

The level of available PPE was considered high (4–6 score, 6 is completely available) by 80% of HCWs. This finding highlights the UAE health policies and actions taken to ensure the provision of medical supplies at the beginning of the pandemic even before the first case in the country [8]. In contrast, the availability of PPEs was considered sufficient by only 21% of HCWs in Portugal [27]. 

The UAE HCWs were largely monitoring themselves daily for COVID-19 symptoms, as it was advised by the international and local health authorities. The UAE local health authorities implemented weekly or bi-weekly mandatory testing for all HCWs [28]. This practice was extremely important to break the chain of community- or hospital-acquired infections, as 12% of the HCWs reported testing positive for SARS-CoV-2. Our self-reported prevalence of SARS-CoV-2 infection was not based on clinical diagnosis from serology, and it was found to be higher than seroprevalence estimates in other countries, such as India (11%) [29], similar to Spain (11.2%) [30], Belgium (12.6%) [31], and New York (13.7%) [32] and lower than China (17.1%) [33], and the United Kingdom (18.0%) [34].

Comparing the findings of the present study with other cross-sectional studies [30] that have been published on psychological distress among HCWs since the beginning of the pandemic in early 2020, it is possible to observe that HCWs in the UAE had lower levels of anxiety and depression compared to the majority of other countries reported but very similar to an umbrella review that reported a prevalence of 24.9% for anxiety and 24.8% for depression [35]. This umbrella review and meta-analysis included seven studies in the meta-analysis from Brazil, China, India, and the United Kingdom [35]. In Egypt, 90.5% of HCWs reported some degree of anxiety. The same study reported that mild anxiety was identified by 40% of HCWs, moderate anxiety by 32%, and severe anxiety by 18.5%. Regarding depression, 94% of the same study population reported mild to severe depression [36]. In Wuhan (China), the prevalence of anxiety was 44.6% and depression 50.4% [37]. In Bangladesh, the prevalence of anxiety was 69.5% and depression 39.5% in a study using HADS [38]. In Portugal, the prevalence of anxiety was 79.1% [27]. In Singapore, the prevalence of anxiety was 10.8% for physicians and nurses, and the prevalence of depression for the same sample was 8.1% [39]. In the present study, physicians had higher prevalence estimates of anxiety compared to nurses (although not statistically significant), which is sometimes the opposite in other settings, like in Turkey, where nurses reported higher levels of anxiety [21]. 

The present study’s findings for moderate-to-high levels of emotional exhaustion and depersonalization are similar to the reported ranges and scores of other studies. An international survey with 184 HCWs from 45 countries from 5 continents reported moderate-to-high emotional exhaustion in 56.0% and moderate-to-high in 48.9% of HCWs [19].

A systematic review of burnout in Arab countries pre-COVID identified 19 studies reporting the prevalence of the three components of burnout [40]. Emotional exhaustion ranged from 20.0 to 81.0%; depersonalization ranged from 9.2 to 80.0%, and personal achievement ranged from 13.3 to 85.8% in the studies included. However, this review did not include any studies from the UAE, as there were none in 2017; however, it reports burnout in HCWs in other Middle Eastern countries: Saudi Arabia, Bahrain, Lebanon, Palestine and Egypt, Jordan, and Yemen [40]. A second systematic review of burnout in HCWs in the Middle East included 54 studies for physicians, 53 studies for nurses, and seven studies for medical residents reported a prevalence ranging from 13 to 85% [41]. 

The only study of burnout in the UAE that we were able to find, also included in the aforementioned systematic review [41], identified moderate-to-high levels of emotional exhaustion in 76%, high depersonalization in 84%, and low personal achievement in 70% of UAE medical residents [14]. Our results in the HCW population (not in trainees) are substantially lower, reflecting better mental health, specifically during the COVID-19 pandemic. This may suggest that experienced HCWs are more resilient and cope better with extreme situations in comparison with less experienced HCWs; this assumption is aligned with recent findings from a similar study in China [37]. Moreover, another recent systematic review identified that the levels of mental health issues in HCWs and the general population differed by only 1% (34% and 33%, respectively) [42]. On this point, there are distinct findings highlighted by research: on one hand, HCWs may be more susceptible to stress, burnout, fear of being infected and of infecting their own family members, as well as of experiencing sleep and emotional disturbances [37,42]. On the other hand, a study from Singapore at the beginning of the pandemic reported that physicians and nurses were more likely to cope better with stressful health events and show more resilience compared with allied healthcare professionals, pharmacists, and lab technicians; again, this varied with seniority [39]. 

### 4.3. Occupational Health Implications of Findings

HCWs have identified the need for better occupational health services focusing on mental health [37]. Continuous mental health services for staff are necessary, and the pandemic highlighted this need that was already felt before the pandemic. To tackle this challenge, lessons learned from the situation should be taken. Mental health specialists have advocated that mental health services should be integrated into occupational health with the establishment of community mental health teams providing both face-to-face and online health services for HCWs, especially during potentially stressful working conditions, such as the COVID-19 pandemic [27,43,44]. 

### 4.4. Strengths, Limitations, and Future Research 

One major strength of this study was the use of validated and widely used scales to identify anxiety, depression, and burnout—the Hospital Anxiety and Depression Scale and the Maslach Burnout Inventory. Moreover, the Cronbach alpha coefficients showed them to be reliable in our sample. These data collection tools made comparisons possible with other international studies before and during the pandemic [19,26]. However, the burnout, anxiety, and depression scores were based on the self-reported answers from the HCWs and cannot be comparable with data from medical diagnoses of these conditions.

Our study design attempted to recruit a representative sample of HCWs from the six major health care providers in the UAE, including publicly funded healthcare and private institutions. However, only four agreed to participate in the study, which might have introduced a selection bias, and the study sample may not have been representative of the entire HCWs population in the UAE. The majority of the study population were nurses (78%), which is in accordance with the distribution of nurses and physicians in Dubai, 67% and 32%, respectively [45], and with a recent study in Abu Dhabi, where 62% of HCWs were nurses, and 20% were physicians [46]. The study also had a higher proportion of females compared to males; some similar studies acknowledge this as a limitation [21], although the natural distribution of occupations in the healthcare sector have been occupied by females, especially nursing. Although we were not able to estimate the proportions for the country in terms of sex, age, occupation, and emirate distribution of HCWs, we have performed the analysis of the study sample as a whole, heterogeneous group. Moreover, the primary inclusion criteria was any HCWs involved in the care of COVID-19 patients, and three-quarters of our study sample (*n* = 1290) were nurses (78.1%; *n* = 1007), and the majority of nurses were female (only *n* = 157 or 15.6% of nurses were male; data not shown), and nurses were more likely to come into contact with or be exposed to COVID-19 patients, which explains the predominance of nurses and females in our study sample. Finally, the nature of the cross-sectional study design needs to be mentioned as well, as it lacks explanations of the impact of the different variables considered on the level of anxiety, depression, and burnout due to no temporality between exposure and outcome. 

Several studies have been published on the same topic, but the data collection periods vary, and for this reason, we highlight here that the comparisons made were done by the type of study and not study period, where the different stages and waves of the pandemic might have had an impact on the anxiety, depression, and burnout levels of the HCWs included in those studies [13,21,27,36,47]. 

Future research on this topic using longitudinal designs would be needed to identify the protective and risk factors for anxiety, depression, and burnout. Additional variables might need to be considered, as years of experience, resilience, being a parent, and family support of HCWs have also been linked to the occurrence of the psychiatric symptoms in previous research [2,37,48].

## 5. Conclusions

HCW’s health should be protected by occupational health services to ensure that they can provide high-quality standard of care to all patients. The HCWs sample represented in the study reporting not having musculoskeletal pain, having performed physical activity, and higher scores of available PPE reported lower scores of anxiety, depression, and burnout. The majority of the study population were female nurses in their thirties, and these findings are fundamental to understand thee sociodemographic characteristics, lifestyle behaviors, and mindsets related to their health, musculoskeletal pain, anxiety, depression, and burnout. The study findings can be used to help formulate better health policies focusing on encouraging physical activity, monitoring the number of night shifts per week, resilience training, and occupational health services focusing on physical and mental health.

## Figures and Tables

**Table 1:** Healthcare worker’s characteristics.

Variables	*n* (%)
Sex	
Male	283 (21.9%)
Female	1007 (78.1%)
Age	
20–29 years old	150 (11.6%)
30–39 years old	662 (51.3%)
40–49 years old	322 (25.0%)
50–59 years old	141 (10.9%)
60 or more years old	15 (1.2%)
Occupation	
Physician	165 (13.0%)
Nurse	1005 (77.9%)
Allied healthcare professional	65 (5.0%)
Lab technician	17 (1.3%)
Not specified	38 (2.9%)
Healthcare Sector	
Publicly funded	1147 (88.9%)
Private sector	143 (11.1%)
Emirate	
Abu Dhabi	100 (7.8%)
Dubai	900 (69.8%)
Northern Emirates	290 (22.5%)

**Table 2:** Cross-tabulations for anxiety and depression by healthcare workers characteristics.

Variables	Anxiety (*n*)	*p*-Value	Depression (*n*)	*p*-Value
Sex				
Male	78	0.732	77	0.764
Female	288		265	
Occupation				
Nurse	271	0.045	246	0.005
Physician	60		59	
Others	35		37	
Healthcare Sector				
Public	333	0.136	309	0.324
Private	33		33	
Hours in Night Shifts				
Less than 9	143	<0.001	118	0.005
Between 9–18	116		126	
More than 18	107		98	
Sleeping Hours				
Less than 6	183	<0.001	163	<0.001
Between 6–8	161		160	
More than 8	22		19	
Physical Fatigue				
0	10	<0.001	9	<0.001
1	11		13	
2	22		28	
3	60		60	
4	94		79	
5	76		77	
6	93		76	
Physical Activity Last Week				
Almost everyday	37	<0.001	30	<0.001
Everyday	5		6	
Less than 2 times	146		128	
Never	178		178	
Adequate Level of PPE at Workplace				
0	5	<0.001	8	<0.0010
1	19		17	
2	25		24	
3	43		38	
4	66		60	
5	85		89	
6	123		106	
Musculoskeletal Pain				
Yes	251	<0.001	243	<0.001
No	115		99	

**Table 3:** Logistic regression models for the Hospital Anxiety and Depression Scale.

	HADS-A			HADS-D		
	aOR	95% C.I.		aOR	95% C.I.	
		Lower	Upper		Lower	Upper
Occupation						
Others	Ref.			Ref.		
Nurse	0.792	0.496	1.264	0.572	0.362	0.904
Physician	1.328	0.757	2.329	1.056	0.608	1.836
Sleeping Hours						
Less than 6 h	Ref.			Ref.		
Between 6–8 h	0.622	0.468	0.827	0.802	0.6	1.071
More than 8 h	0.404	0.237	0.689	0.45	0.258	0.785
Physical Fatigue						
0	Ref.			Ref.		
1	1.778	0.815	3.879	2.864	1.27	6.459
2	2.464	1.162	5.222	2.459	1.088	5.557
3	3.156	1.582	6.295	3.861	1.83	8.148
4	3.512	1.748	7.059	3.907	1.832	8.332
5	4.86	2.391	9.877	4.862	2.256	10.48
6	12.187	5.854	25.369	9.289	4.236	20.371
Physical Activity Last Week						
Almost everyday	Ref.			Ref.		
Everyday	0.534	0.191	1.492	0.943	0.357	2.491
Less than 2 times	1.427	0.923	2.204	1.621	1.02	2.577
Never	1.719	1.111	2.659	2.375	1.499	3.763
Musculoskeletal Pain						
Yes (vs. No)	1.424	1.068	1.898	1.713	1.276	2.3

aOR, adjusted odds ratio for adequate level of PPE at workplace.

**Table 4:** Scores of burnout, per domain, among healthcare workers.

Variables	Emotional Exhaustion (*n*)			*p*-Value	Depersonalization (*n*)			*p*-Value	Personal Achievement			*p*-Value
	Low	Moderate	High		Low	Moderate	High		Low	Moderate	High	
Sex												
Male	117	127	39	0.113	192	78	13	0.068	5	75	203	0.340
Female	486	392	129		752	222	33		10	300	697	
Occupation												
Nurse	486	395	123	0.100	747	225	33	0.047	11	300	694	0.004
Physician	62	77	26		113	48	27		3	57	105	
Others	54	47	19		84	27	9		1	18	101	
Healthcare Sector												
Public	529	468	150	0.423	840	269	38	0.362	15	337	795	0.283
Private	74	51	18		104	31	8		0	38	105	
Hours in Night Shifts												
Less than 9	268	187	42	<0.001	388	96	13	0.002	10	126	361	<0.001
Between 9–18	236	201	60		364	116	17		2	148	347	
More than 18	99	131	66		192	88	16		3	101	292	
Sleeping Hours												
Less than 6	138	213	91	<0.001	291	126	25	<0.001	7	150	285	<0.001
Between 6–8	362	270	62		529	146	19		6	198	490	
More than 8	103	36	15		124	28	2		2	27	125	
Physical Fatigue												
0	123	16	1	<0.001	123	16	2	<0.001	3	18	119	<0.001
1	94	17	1		104	8	0		0	10	102	
2	66	36	9		84	25	3		2	30	79	
3	127	108	18		185	63	6		3	82	168	
4	115	150	25		218	67	6		2	108	180	
5	55	124	47		148	65	13		1	68	157	
6	23	68	67		82	56	20		4	59	95	
Physical Activity Last Week												
Almost everyday	133	65	10	<0.001	175	28	5	<0.001	2	46	160	<0.001
Everyday	33	16	4		44	6	3		0	6	47	
Less than 2 times	249	214	63		398	114	14		4	139	383	
Never	188	224	91		327	152	24		9	184	310	
Adequate Level of PPE at the Workplace												
0	5	1	6	<0.001	5	7	0	<0.001	0	4	8	<0.001
1	9	17	10		22	11	3		1	15	20	
2	12	24	13		23	21	5		2	21	26	
3	43	65	18		83	38	5		1	57	68	
4	89	83	35		151	49	7		4	75	128	
5	157	140	39		240	83	13		2	93	241	
6	288	189	47		420	91	13		5	110	409	
Musculoskeletal Pain												
Yes	240	315	144	<0.001	451	210	46	<0.001	5	234	460	<0.001
No	363	204	24		493	90	8		10	141	440	

**Table 5:** Regression models for the overall scores of burnout components.

				95% C.I.	
Burnout Overall Score	Variables	Beta	*p*-Value	Lower	Upper
Emotional exhaustion	Physical fatigue	0.415	<0.001	2.754	3.462
	Musculoskeletal pain	0.151	<0.001	2.857	5.428
	Adequate level of PPE at workplace	−0.139	<0.001	−1.805	−0.932
	Physical activity	0.108	<0.001	0.834	2.021
	Sleeping hours	−0.107	<0.001	−3.266	−1.314
	Sex	−0.069	0.002	−3.722	−0.818
	Hours In night shifts	0.064	0.006	0.328	1.941
Depersonalization	Physical fatigue	0.262	<0.001	0.712	1.084
	Musculoskeletal pain	0.114	<0.001	0.760	2.113
	Adequate level of PPE at workplace	−0.106	<0.001	−0.708	−0.248
	Sex	−0.089	0.001	−2.113	−0.587
	Physical activity	0.073	0.006	0.128	0.754
	Age	−0.060	0.021	−0.795	−0.064
	Sleeping hours	−0.059	0.027	−1.095	−0.067
Personal achievement	Adequate level of PPE at workplace	0.190	<0.001	0.748	1.318
	Age	0.154	<0.001	0.885	1.799
	Physical fatigue	−0.119	<0.001	−0.716	−0.270
	Occupation	−0.102	<0.001	−2.485	−0.806
	Physical activity	−0.070	0.010	−0.894	−0.122
	Sleeping hours	0.061	0.024	0.094	1.356

## Data Availability

The data presented in this study are available on request from the corresponding author. The data are not publicly available due to privacy issue.
